# Supplementary material for: Alternative proteoforms and proteoform-dependent assemblies in humans and plants
Source: Mol Syst Biol. 2024 Jun 25;20(8):933–51. doi: 10.1038/s44320-024-00048-3 (PMC11297038; doi:10.1038/s44320-024-00048-3)
Supplement: Supplementary file 4 — Expanded View Figure [file 44320_2024_48_MOESM4_ESM.pdf]

# Expanded View Figure

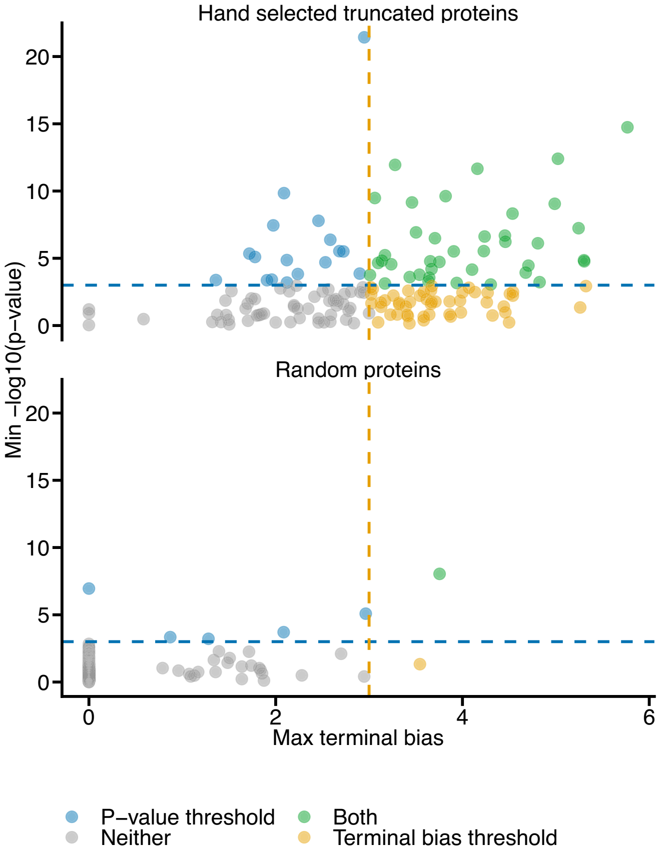


**Figure EV1. Both maximum terminal bias score and minimum p-values prioritize proteins with apparent short proteoforms relative to randomly selected proteins.**

Proteins in green are prioritized by both methods, proteins in blue are prioritized by the *P* value threshold only, proteins in yellow are prioritized by the terminal bias threshold only, and those in gray by neither method.

© The Author(s)
